# Teachers’ gaze over space and time in a real-world classroom

**DOI:** 10.16910/jemr.13.4.1

**Published:** 2018-09-14

**Authors:** Zuzana Smidekova, Miroslav Janik, Eva Minarikova, Kenneth Holmqvist

**Affiliations:** Department of Educational Sciences, HUME Lab – Experimental Humanities Laboratory, Masaryk University, Czech Republic; Institute for Research in School Education, Masaryk University, Czech Republic; Department of Psychology, Copernicus University, Poland; Department of Psychology, Universität Regensburg, Germany; Faculty of Arts, Masaryk University, Czech Republic

**Keywords:** Eye movement, eye tracking, attention, region of interest, individual differences, gaze

## Abstract

Reading students’ faces and their body language, checking their worksheets, and keeping eye contact is a key trait of teacher competence. The new technology of mobile eye-tracking provides researchers with possibilities to explore teaching from the viewpoint of teacher gaze, but also introduces many new method questions. This study had the primary aim to investigate teachers´ attention distribution over space: the number and durations of several types of their gazes, and how their gaze depends on the factors of students´ gender, achievement, and position in the classroom. Results show that teacher gaze was distributed unevenly across both space and time. Teachers looked at the most-watched students 3-8 times more often than at the least-watched ones. Students sitting in the first row and the middle section received significantly more gaze than those sitting outside this zone. All three teachers made more single gaze visits - looking at the students but making no eye contact - than mutual gazes or student material gazes. The three teachers’ gaze distribution also varied substantially from lesson to lesson. Our results are important for understanding teacher behavior in real classrooms, but also point to the relevance of appropriate method design in future classroom studies with eye-tracking.

## Introduction

Teaching is a demanding profession. Teaching situations are characterized by simultaneity, multidimensionality, unpredictability, and immediacy (1). When researching what happens in the classroom, it is of great importance to understand how teachers orientate themselves and attend to the plethora of stimuli, as a means to further our knowledge of what and whom teachers prioritize. Up until recently, our only possibility to investigate what and whom teachers notice in classrooms was to ask them: Verbal data has traditionally been the empirical foundation for research on teachers’ attention distribution, in combination with observing them during teaching, or from video recordings of the classroom (2, 3, 4). But recently, with the rise of modern eye-tracking glasses, researchers can explore teachers’ gaze from detailed online measurements in the classroom (5, 6, 7, 8, 9, 10, 11, 12, 13, 14, 15).

### Teachers’ gaze distribution

Teachers’ gaze distribution can be described by a gaze proportion across the people and objects in the classroom. This proportion is based on measurements of how many times and for how long a person looks at a specific region relative to alternative regions. To maximize ecological validity of research on teachers’ gaze, we need to explore what expert teachers prioritize in their everyday teaching practice in actual classrooms. When doing so, some studies have found that experienced teachers demonstrate greater equity in their gaze across students than novice teachers (5, 6).

In our study, we focus on three factors that potentially affect teachers’ visual attention in the classroom: student gender, student achievement, and overall classroom composition. The selection of the factors student gender and achievement was based on Howe and Abedin’s (16) review of 92 studies on classroom dialogue, which shows that 45 of those studies found that students’ gender influenced the dialogue between the teacher and the students. 17 out of the 92 studies showed the same for achievement.

Teachers may believe that they treat girls and boys equally, but many classroom observations suggest that this is not often the case (17, 18, 19 ,20). Two meta-analytic studies conducted by Kelly (21) and Jones and Dindia (22) indicate that teachers interact more with male students than with female students. It is argued that teachers interact on average 10 – 30% more with boys than with girls (23), and this difference has been found to depend on the student’s grade and the teacher’s personality (24). Male students are consistently more outspoken as well as more unreserved compared to female students (25). It has been found that boys receive more responses from teachers (both praise and reprimand) than girls (19, 26, 27, 28).

There are several possible reasons behind the observed gender preferences by teachers. One possible reason can be related to the greater assertiveness of boys. That is, if boys are speaking up more frequently in discussions or at other times, teachers may feel implored to pay more attention to them (29, 30). Another possibility is that some teachers may feel that boys are more likely to act mischievously. Consequently, teachers may interact with boys more frequently to keep them focused on the task at hand. Still another possibility is that boys, compared to girls, may interact in a wider variety of styles and situations. In other words, there may be a richer range of opportunities for teachers to interact with boys (31). On the other hand, several studies highlight a higher level of negative attention and tendency to criticize boys in the classroom (32, 33, 34). Despite the growing evidence on teacher-student gender differentiated interaction, it can be assumed that teachers are not aware of taking students’ gender into account when teaching (35, 36, 37).

A student’s achievement can be seen as another factor affecting the amount of attention given to the individual student. It has been found that low achieving boys receive less feedback and fewer opportunities to respond from the teacher, which stands in contradiction to boys, in general, getting more teacher attention (38). Higher achieving students acquire not only more attention (39) but also more differentiated attention (40). Conversely, teachers wait a shorter time for the responses from low achievers, provide less guidance to elicit the correct response from them, and ask less demanding questions to low achievers. It was found that teachers who believed they were interacting with high achieving students smiled and nodded their heads more often than teachers interacting with low achieving students. Teachers also leaned towards high achievers and looked high achievers in the eyes more frequently (41). Overall, the general demand on low achievers is less than on high achievers. Later on, this was emphasized in studies on teacher noticing (professional vision), especially in terms of developing teacher noticing for equitable practices (42).

A large video study from the 1960s (43) showed that apart from the subject being taught, the student’s age, and the teacher’s age or gender, there were physical positions in the classroom identified, where communication was more common than elsewhere. The zone with more communication had the shape of a reversed letter T: More interaction took place at the front and middle part of the classroom. In our study, we will refer to this space as the T-Zone. The existence of the T-Zone was confirmed by further studies (44, 45, 46, 47, 48, 49, 50, 51, 52, 53). Moreover, other studies have found out that students sitting in this zone get better grades and like the teacher more (54).

To our knowledge, factors of gender, achievement, and position in the classroom have not been examined yet by mobile eye-tracking technology that has the potential to provide more reliable outcomes than observation or using classroom videos as prompt for verbal data.

### Why study eye contact in the classroom?

Critical communication variables present in teacher-student relationships are represented by nonverbal behavior (55, 56) as well as gaze direction, which is considered to be directly related to individual thoughts since it was evidenced by the eye-mind hypothesis (57) studies (58, 59, 60, 61, 62). Huang (63) and Ledbury, White and Darn (64) suggest that teachers watch students and listen to them while they perform tasks, particularly when they look for students’ signs of being bored or being lost. Thus, eye contact does not have to be considered as a tool of the teachers to convey messages, but as a method to interpret the messages students can display nonverbally via their eyes, mimics, and gestures.

It has been claimed that real communication between two persons begins only when eye contact is established (65, 66, 67 ,68). According to Gower and Walters (69), the main use of eye contact in the classroom consists of showing a student, who is talking, that the teacher is taking notice; checking that everyone is concentrating and indicating to a student that the teacher wants to talk to him. Furthermore, eye contact is used to encourage contributions when the student is trying to elicit ideas (70, 71, 72), to hold the attention of students not being addressed and to maintain their attention (73). Eye contact can also be interpreted as a sign of teacher approval or disapproval, affecting motivation (74).

Many benefits to eye contact in the classroom have been pointed out in previous research. Fry and Smith (75) found that college students performed better on a coding task when they received instructions from a teacher gazing at them compared to a non-gazing teacher. Primary school children had a greater recall for stories that were read by a gazing rather than non-gazing teacher (74). Breed and Colaiuta (44) found a positive correlation between the amount of student eye contact with an instructor and student comprehension. Higher test scores were associated with increased time looking at the instructor during discussions and less time looking elsewhere about the room. Eye contact also influences the quality of communication during teaching (76, 77). It has been shown that students participate more in a seminar when they could make eye contact with the instructor. An initial look increases the probability of an ensuing conversation and decreases the incidence of no talking (78). The effect of direct gaze on performance has been explained by attentional processes, suggesting that direct gaze helps to retain attention in the task (74, 79, 80, 81, 82).

Since eye contact and facial expressions are considered signs for teachers’ self-confidence, they have an impact on teachers’ credibility and trustworthiness. A teacher who never looks students in the eye seems to lack confidence and gives the students a sense of insecurity (69, 83). Eye contact with the students contributes to teachers receiving higher student evaluations (84). Breed and Colaiuta (44) found that college instructors were not only better liked, but also produced superior student performance when they gazed at students using longer gazes during their lectures. Numerous studies have found that mutual gaze has a profound impact on cognition and emotion across the lifespan, a phenomenon referred to as the ‘eye contact effect’ (85). Research in social psychology has documented adult gaze to be part of a system of natural pedagogy whereby teachers signal behaviours through eye contact which function as part of an innate framework by which infants, even newborns, learn (86, 87, 88). Research has shown that eye contact can be viewed as a welcoming signal that encourages approach, whereas averted gaze discourages it (78, 89) as well as increases rapport/relationship (64). When a teacher visually neglects a student, he interprets that as the teacher having no expectations for him/her, and we know that students’ performance is directly influenced by teacher’s expectations (the Rosenthal effect (90)). Positive interactions are characterized by an increased frequency and duration of eye contact (91). Although prior research has demonstrated that speakers who direct more gaze toward their audience are perceived as more persuasive, likeable, and competent by listeners (92, 93), there has been more recent work (94), claiming that more eye contact between the listener and speaker during persuasive communication predicts less attitude change in the direction advocated.

A sustained gaze from the student to the teacher has been taken to indicate interest (95), but instructors also quite often notice how students avoid eye contact. Knapp and Hall (96) confirm the most common interpretation of avoiding eye contact is that the student does not know the answer to a question. Waxer (97) points out that students will avoid eye contact when they simply dislike the subject matter and when they are disinterested. Students with low self‐esteem or evasive students are also likely to avoid eye contact (98, 99). Looking away from a teacher’s face during demanding cognitive activity can help students to answer cognitively challenging questions (100, 101). While such *gaze aversion* is used far less by 5-year-old school children, its use increases dramatically during the first years of primary education, reaching adult levels by 8 years of age (102).

Evidence based on eye-tracking in static social scenes in laboratory studies postulates a tendency of participants to fixate the eyes of any faces (59, 103, 104). However, when people are physically present, we look into their eyes less frequently (105, 106). This means, that whatever evidence we can draw from lab-based experiments, we cannot take for granted that they translate to the real-world situations (107). Insights gained from studies using natural settings are not only helpful but also essential to properly investigate social attention and the factors that affect eye contact (108).

### Research questions

The main aim of this exploratory study is to investigate the gaze distribution of three experienced teachers towards the students during four lessons each in real-world classrooms. The data analysis was supplemented with inferential tests. Four questions were studied:

Firstly, what is the level of equality or inequality in the gaze distribution towards each student in the classroom?

Secondly, do teachers’ gaze distributions depend on specific characteristics of their students, known from previous research: Will teachers gaze more on boys in comparison to girls, will more gaze go to high achievers than to low achievers, and will more gaze go to the students sitting in the T-Zone than to those sitting outside the T-Zone?

Thirdly, what is the frequency and duration of teacher gazes, for mutual gaze (when the teachers were maintaining eye contact with the students), for single gaze (teachers were looking at the students without the students looking back) and for gazes on students teaching material (textbooks and notes)?

Fourth, how does that gaze behavior change over time? Are teachers consistent in their gaze behavior over four subsequent lessons with the same class?

## Methods

### Participants

For this study, we used gaze data from three experienced practicing female teachers (at least 5 years of teaching practice) of English as a foreign language. All three teachers work in the same primary and lower secondary school located in a small town (approx. 4 000 inhabitants) in the Southern Moravia Region of the Czech Republic. Participating teachers were chosen via recommendation of the school headmaster. Each teacher selected one class to participate with her in the study. The traits of the teachers and their classes are provided in Table 1. All three teachers had very similar characteristics in terms of age, education, and teaching experience, which helped us to form what we considered a homogeneous teacher sample for our study. All the teachers had normal or corrected-to-normal vision. The teachers were asked to provide information about selected student characteristics. This information is used in conjunction with the eye-tracking data. Student’s achievement is based on their overall grade in the last semester. In the Czech educational system, grades 1 to 5 can be awarded, 1 being the best, 5 being failed level. We formed the groups ´high´= 1 or 2 and ´low´ = 3 or 4. No failed level (5) student was in any of the participating classes.

**Table 1:** Characteristics of teachers and their classes.

Teachers	T1	T2	T3
Education	Teacher Education, MA degree	Teacher Education, MA degree	Teacher Education, MA degree
Teaching experience at schools (English as foreign language)	12 years	10 years	6 years
Classroom of participation	Grade 5 (students aged 10 to 11)	Grade 6 (students aged 11 to 12)	Grade 6 (students aged 11 to 12)
Number of students and their characteristics	16 students (5 girls, 11 boys; 12 high achievers; 4 low achievers)	24 students (13 girls, 11 boys; 15 high achievers; 9 low achievers)	16 students (8 girls, 8 boys; 11 high achievers; 5 low achievers)

Since we could not experimentally control and balance the number of boys and girls, or low achieving and high achieving students and their position in the classroom, their proportions by each teacher are presented in Table 2. There are some notable imbalances. For instance, in the classroom of teacher 1, all of the low achieving students were sitting in the T-Zone area. In the classroom of teacher 3, mostly boys were sitting in the T-Zone area while girls were outside of the T-Zone area. The majority of low achieving students were boys for all three teachers.

**Table 2:** The volume of students according to the position in the classroom (T-Zone), Gender and Achievement.

		Gender					
		T1		T2		T3	
		M	F	M	F	M	F
Achievement	Low	3	1	5	4	4	1
	High	8	4	6	9	4	7
		Gender					
		T1		T2		T3	
		M	F	M	F	M	F
T-Zone	In	7	2	5	7	6	2
	Out	4	3	6	6	2	6
		T-Zone					
		T1		T2		T3	
		In	Out	In	Out	In	Out
Achievement	Low	4	0	5	4	4	1
	High	5	7	7	8	4	7

### Lessons

Altogether 12 lessons of English as a foreign language (4 lessons by each teacher) were collected. Each lesson was approximately 45 minutes long, yielding a total of 9 hours of data. This study, aiming to study teacher gaze during real class settings, allowed teachers to work with their classes as usual. They could move across the classroom and exhibit their everyday teaching behaviors. The only recommendations given to the teachers were to lead the lessons with teaching that required communicative situations, to reduce written examination of students during the recorded lessons, and to keep the students sitting at their usual places in the classroom. The seating configuration for the students was arranged by the teachers. Students were sitting in this arrangement for a longer period before our data collection started. The classroom seating arrangement in all three cases consisted of a pair pod setup, in which students face the teacher with their backs to other students. This seating arrangement is still very often used at Czech primary and lower secondary schools.

As for the lesson content, teacher 1 used mostly whole class work with activization elements (games, activities done outside students’ desks) with individual work mixed in. On the other hand, teacher 2 centers her lessons around pair and group work, using individual work as preparation for it and whole class work mostly as its introduction and wrap up. For teacher 3, a large proportion of whole class work was typical, while individual or pair work appeared mostly in phases of completing tasks. Despite our recommendation to include communicative tasks, all teachers emphasized grammar and vocabulary work through their choice of activities and through their remedial work; teacher 2 consistently included information gap activities, albeit aimed at practicing grammar and vocabulary. The lessons were fairly consistent in structure, although the proportion of the three types of work varied slightly.

To the best of our knowledge, no previous studies have recorded more than one full lesson of eye-movement data from the same teacher. If there is substantial variation in gaze behavior between lessons, recording a single lesson per teacher runs the risk of not describing the typical behavior of that teacher. For this reason, we have selected to record four full lessons per teacher, so we can investigate the consistency of teacher gaze behavior over time.

### Apparatus and data collection

During the lessons, teachers wore SMI Eye Tracking Glasses 2 Wireless (ETG; 60Hz). Prior to the start of data collection, all teachers tried and tested the glasses. Before each recording, a 3-pt calibration was used to ensure good calibration accuracy. Participants were asked to fixate on 3 locations in the classroom (within a participant’s field of view). The eye-tracker also yielded audio recordings. The data was collected during autumn 2018. The process consisted of four individual recordings of subsequent lessons for each teacher, followed by four interviews regarding the students’ characteristics, teachers’ professional background and teachers' impressions of the recorded lesson. The feedback on the possible interference of eye-tracking glasses with student and teacher behaviour was discussed.

Before data collection began, consent and agreement forms signed by the teachers and by all the students’ parents were obtained, which were in agreement with the GDPR (General Data Protection Regulation), the European Data Protection Regulation (EU) 2016/679, which is applicable as of May 25th, 2018 in all member states to harmonize data privacy laws across Europe. The study was approved by the Research Ethics Committee — Masaryk University.

### Coding and data processing

**Figure 1:**
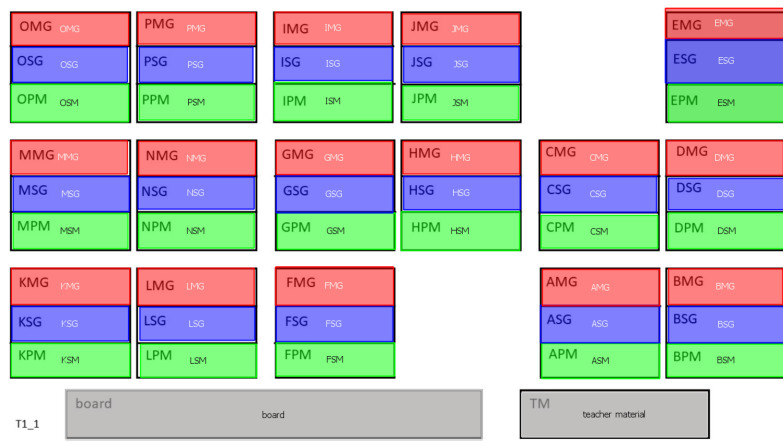
Reference view image with pre-drawn AOIs in SMI Semantic Gaze Mapping in BeGaze software.

All the recorded data were uploaded into the SMI BeGaze analysis software, in which a reference view image was made with the configuration of the classroom arrangement for each teacher and each lesson. Figure 1 shows the prearranged reference view image for the first lesson of teacher 1. In the next step, areas of interest (AOIs) were drawn over the reference view images. Student-related AOIs were made not only for each of the students sitting in the classroom but for three possible modes per student. Each student had a red colored AOI named *mutual gaze*, representing cases of mutual eye contact between the teacher and the student. The blue colored AOI named *single gaze* was used when the teacher was looking at the student (face or upper body area), but the student was looking somewhere else. When the teacher was looking at the student’s material (book, notebook, worksheet), we used a green colored AOI named *student material*. We use the short-hand MG, SG, and SM for these three cases (see Figure 1). Eye-tracking data was then manually coded, fixation by fixation, using the Semantic Gaze Mapping feature of the SMI BeGaze software.

### Eye movement measure considerations

**Figure 2:**
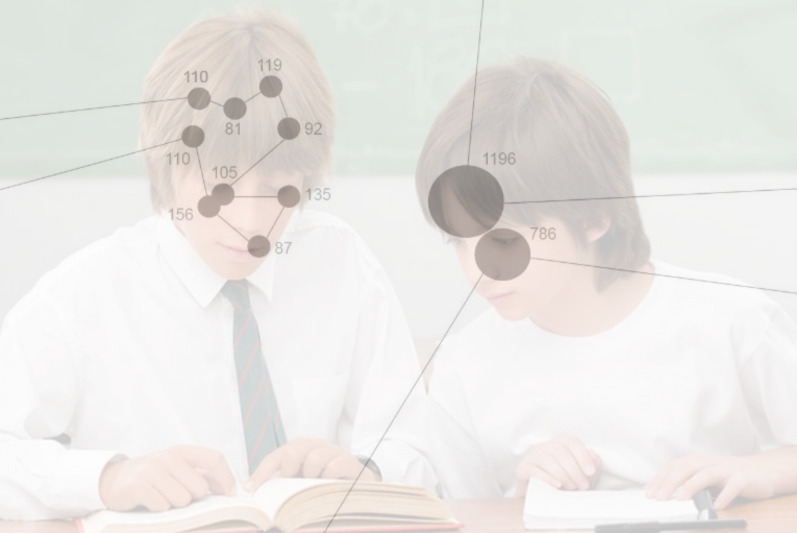
Fixations vs. visits and dwell times.

Previous literature has reported results from classroom recordings using the number of fixations as their fundamental eye-movement measure (5, 7, 8, 10, 11, 15). This choice makes the results dependent on the event detector used for calculating fixations, its ability to compensate for head movements, its sensitivity to noise and the resolution of the eye-tracker. Therefore, we chose to base our results on two less sensitive measures (109), illustrated by Figure 2.

Visits (number during each lesson) – how many times the teacher entered an AOI, or in other words, looked at a student.

Dwell Time (average during each lesson) – for how long the teacher stayed during each visit to an AOI (student).

Figure 2 shows that the student to the left has been visited once, with a total dwell time of 986 milliseconds (ms), divided into 8 fixations. The student on the right has been visited twice: Once, the dwell time was 1196 ms, and the second dwell time was 786 ms. The number of visits (1 on the left, and 2 on the right) in combination with the total duration of each visit (986 ms on the left, and 1196 ms and 786 ms on the right) is a better basis for comparison than the number of fixations.

In Table 4 and Figure 5 below, we make parallel analyses with fixations vs. visits and dwell time and other common eye-movement measures, to illustrate the importance of selecting the appropriate measures for classroom research.

### Analysis of data

All analyses of data were done using R 3.6.1. GINI calculations were made using the function gini from the package “Reldist”. Linear regressions were modeled for the number of visits and average dwell time, using the lm command, with a teacher, type of AOI, gender, achievement, and T-Zone as predictors. Because this is a case study where we do not attempt to generalize over teachers or students, we chose not to model teachers as random factors, and because they constitute AOIs, the students were also not modeled as random factors. Also, we were not looking for interactions, which Table 2 suggests would be caused by natural, unbalanced data. The following was the lm call.

fit <- lm(Visits ~ Teacher + Lesson + Type_of_AOI + Gender + Achievement + T.zone, data = zuz, na.action = na.omit)

## Results

### Variety in the teachers’ gaze distribution

To inspect the variety in teachers’ gaze distribution towards students, we first calculated differences in students’ visits between the most and least visible students. In this part we combine single gaze and mutual gaze AOIs, but do not include student material AOIs, to make results comparable to the two previous studies (5, 6). Table 3 indicates the contrast of numbers of visits to the most and least-watched student in the classroom during each lesson. The mean values of this difference are showing that the smallest difference (38 visits) was found by the teacher 2. For the lessons’ variation, the smallest was observed again by the teacher 2.

**Table 3:** The absolute and relative difference in visits between the most and least attended students most and least attended students.

	T1		T2		T3	
	Abs. diff. Visits	Rel. diff. Visits	Abs. diff. Visits	Rel. diff. Visits	Abs. diff. Visits	Rel. diff. Visits
Lesson 1	71	6.5	38	7.6	136	9.1
Lesson 2	99	3.7	62	10.3	87	3.9
Lesson 3	57	0.8	81	6.8	73	3.2
Lesson 4	83	2.8	66	16.5	87	2.5
MEAN	78	3.5	62	10.3	96	4.5

Table 3 shows that all three teachers look at (visit by gaze) the most attended student 3-8 times more often (around 80 more looks) than the student least looked at during the average lesson. This is an extreme difference and it raises the question of whether teachers are unfair in their distribution of gaze or whether this variation is an adaptation to specific means of managing the classroom.

A complete overview of the equality of gaze distribution across all the students in the classroom (rather than just the most and least visible ones) is provided by calculating GINI coefficients. To describe the distribution of gaze, several measures could be used (range, variance, etc. (109)). The GINI coefficient, frequently used in sociological and economic research as a measure of statistical dispersion, is the most commonly used measurement of inequality. It has been used in previous eye-tracking research (5, 6). The GINI coefficient is a more appropriate measure of inequality of distribution in this case, than range or variance, because the measures for students are not statistically independent. If student A receives a lot of attention from the teacher, less is left to distribute among the others (5). A GINI coefficient of zero expresses perfect equality, where all the students have received the same amount of gaze from the teacher. A GINI coefficient of one expresses maximal inequality among values: One student gets all the attention and all other students are ignored (110, 111).

The calculated GINI coefficients for our data suggest that gaze across all the students in the classroom was not distributed perfectly equally (GINI coefficient of zero): Table 4a indicates that some students or groups of students are obtaining more attention than others. The GINI coefficient values identified were similar among all three teachers.

**Table 4a:** Mean GINI coefficients of mutual gaze and single gaze visits values using data from all four lessons by each teacher.

	T1	T2	T3	MEAN
Visits	0.36	0.37	0.31	0.35

For the purpose of comparing the GINI of visits to those of other measures, in Table 4b we also present GINI coefficients for Average dwells and Fixation durations, two common measures used in classroom research.

**Figure 3:**
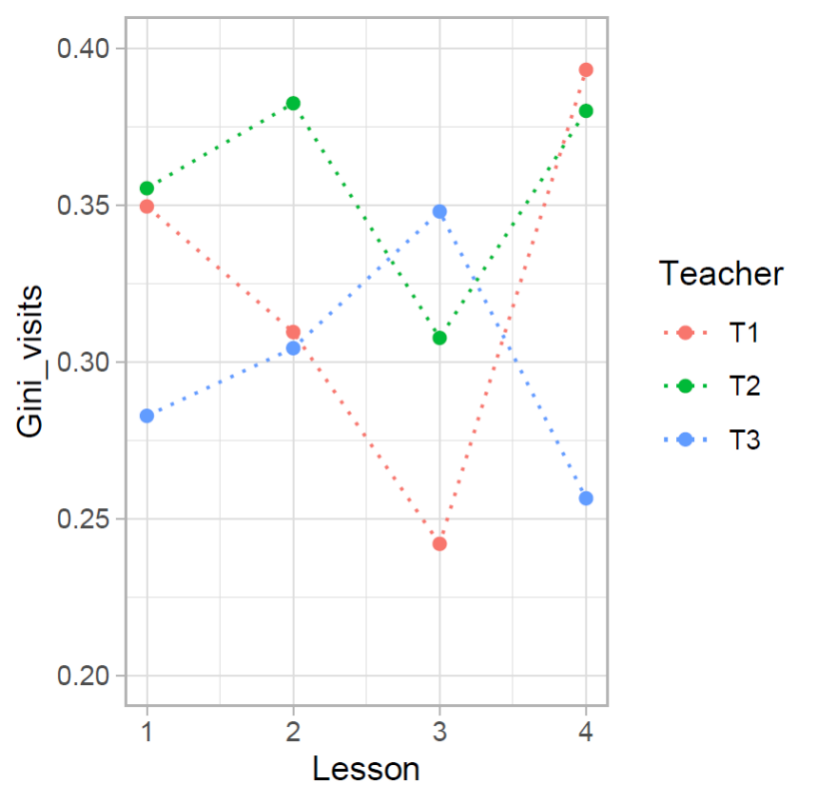
GINI coefficients of mutual gaze and single gaze visits for three teachers over four consecutive lessons.

Irrespective how we measure, the GINI values are similar between teachers, when taken across all four lessons. However, as shown in Figure 3, all teachers’ equality in the distribution of attention fluctuated considerably over the lessons, especially for teacher 1. In other words, the same teacher may behave very differently when we compare different lessons.

**Table 4b:** Mean GINI coefficients of average dwell, number of fixations, relative dwell time and fixation duration values, using mutual gaze and single gaze data from all four lessons by each teacher.

	T1	T2	T3	MEAN
Average dwells	0.19	0.21	0.19	0.20
Number of fixations	0.41	0.43	0.33	0.39
Relative dwell time	0.41	0.44	0.34	0.40
Fixation durations	0.10	0.12	0.08	0.10

Teachers have lower GINI scores for average dwells than for visits, which tells us that they are less unequal with the duration that they spend looking at students. Number of fixation and relative dwell time are amalgamated measures combining visit and average dwell information. Teacher fixation durations do not seem to vary much for different students.

### Gaze distribution with respect to the three characteristics of the students

We have analysed the gaze distribution with respect to the three selected characteristics of the students: their gender, achievement, and position in the classroom.

The linear regression model of the number of visits revealed significant effects in the factors of teachers (F(2)=70.5; p<0.001) and the position in the classroom (referred to as the T-Zone) (F(1)=24.8; p<0.001). No interaction effects were found. Closer inspection showed that teacher 2 visited all students related AOIs less than teacher 1 by 13.6 ± 1.5 visits (t=-9.3; p<0.001). Teacher 3 and teacher 1 visited student’s related areas with similar rates – no significant differences were identified. Overall, there was a significantly higher rate of visits for the T-Zone area by 5.9 ± 1.2 visits compared to outside the zone (t=-5.0; p<0.001).

**Figure 4:**
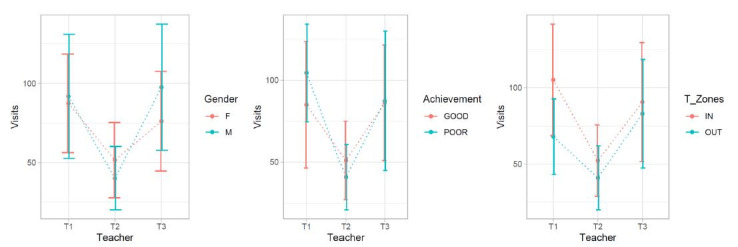
Number of visits to boy and girl students (left); number of visits to high and low achieving students (middle); number of visits to students sitting in and outside the T-Zone (right) for each teacher (mean values of four lessons)

We further inspected trends in gathered data using graphs. There was an apparent trend of more visits inside the T-Zone area, which is reflected in the results of the linear regression. In contrast to earlier findings in literature (21, 22, 23), our Figure 4 did not show any specific tendency to pay more attention to either boys or girls and no tendency to pay more attention to either high or low achieving students. Figure 4 also shows a large variability within each teacher, which is not explained by the three factors Gender, Achievement and T-Zone, when data are collapsed over the whole lessons.

### Frequency and duration of mutual gaze, single gaze, and gaze on student material

**Figure 5a:**
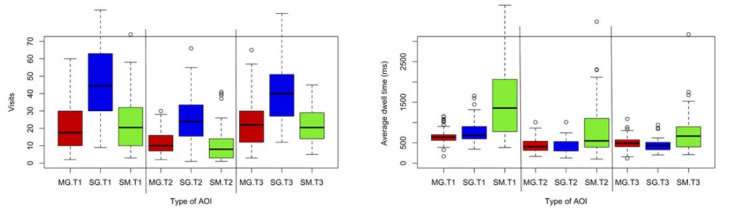
Frequency (left) and duration (right) of visits. Single gaze visits are most common for all three teachers. Student material gazes have the longest visit durations for all three teachers (the data includes all four lessons).

We argued above that the measures we use, visits and average dwell time, provide more beneficial information than the more commonly used measure: number of fixations. In Figure 5a, we plot comparisons of the frequency (left) and duration (right) of all the visits (gazes/looks) at students throughout all four lessons, reported separately for mutual gaze (MG), single gaze (SG) and gaze on student material (SM). The charts in Figure 5a show that all three teachers made significantly more single gazes (on average 18 more) than any other kind of gaze (t=13.1, p<0.001). The most common behaviour was the teachers looking at the students and students were not looking back to them. The number of mutual gazes (visits/looks) was about the same as the number of looks at student material.

However, the low frequency of student material gazes is strongly contrasted by their longer durations compared to both mutual and single gaze. This difference in frequency and duration between the categories highlights the specific nature of each kind of gaze. Some categories of gaze are frequent and short (single gaze) while others are less frequent and longer (student material).

Maybe surprisingly, the behaviour we see so clearly in Figure 5a will not be visible in studies that use other metrics. In order to demonstrate the importance of measure selection, we have plotted in Figure 5b the same data using common measures from literature. It can be clearly seen that the effects in Figure 5a are no longer visible. This is because of the amalgamating behavior of Number of fixations and Relative dwell time. In Figure 5b, these two measures produce equal bars for the SM and SG AOI groups. Average fixation duration just reflects individual differences in oculomotor control. We know from Figure 5a that fewer but longer SM contrast against more frequent but shorter SG gazes. In Figure 5b this finding is hidden by the choice of measures.

**Figure 5b:**
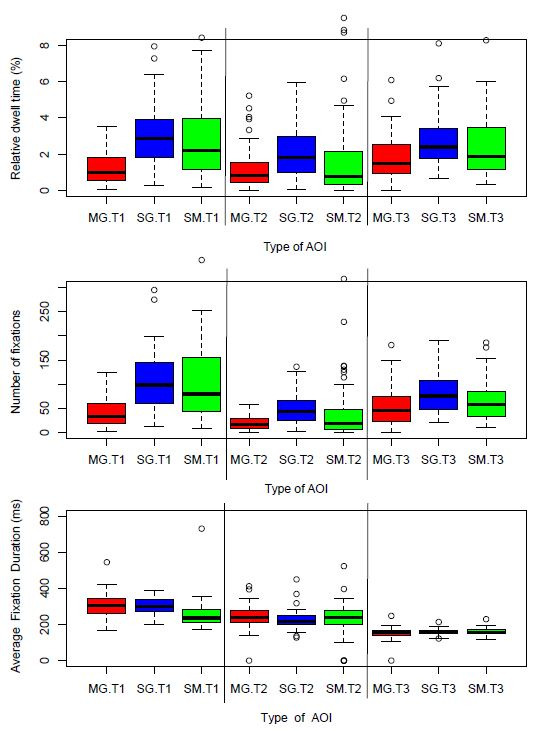
Three measures commonly used in classroom research: Relative dwell time, Number of fixations and Average fixation duration.

### Proportions of dwell time of mutual gaze, single gaze, and student material

**Figure 6:**
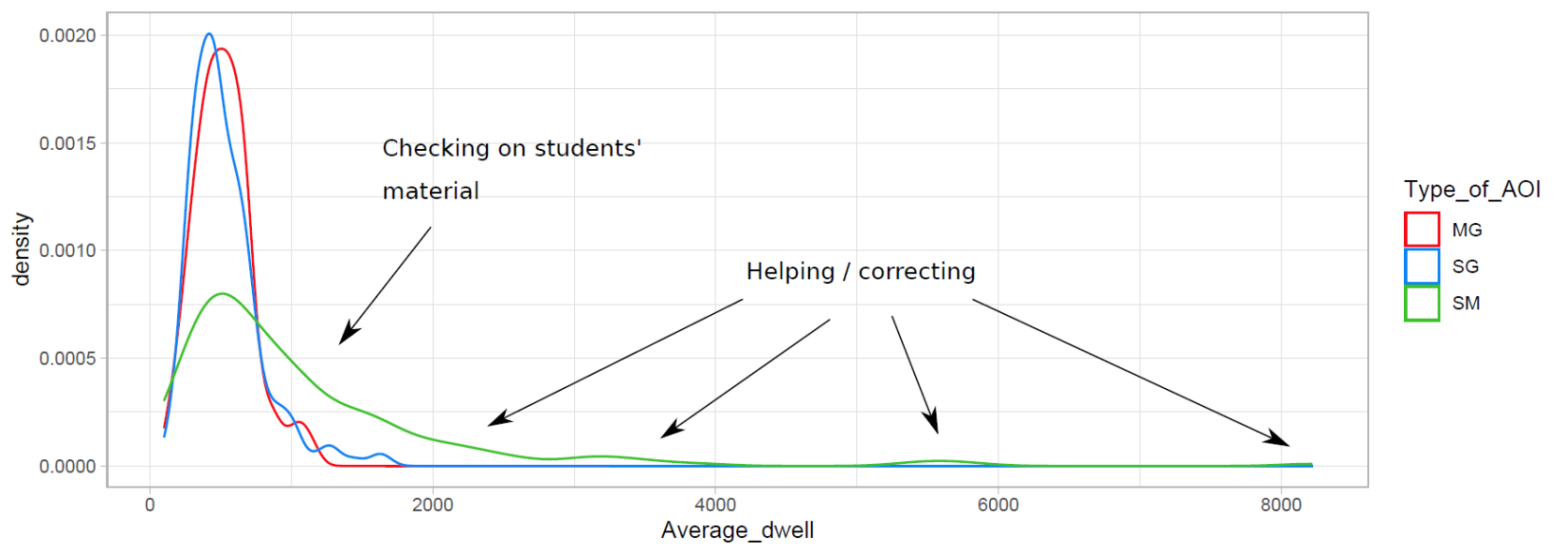
Kernel density plot of dwell time durations of mutual gaze, student material, and single gaze.

The proportion of dwell times and frequency is visualised in Figure 6 which shows that single and mutual gaze have identically short dwell times while the dwell times for the student’s material can be both short and long. This difference suggests a double function of gazes on student material: briefly checking vs. the need for intervention.

### Variety in single gaze, mutual gaze and student material gaze over the 4 lessons

**Figure 7:**
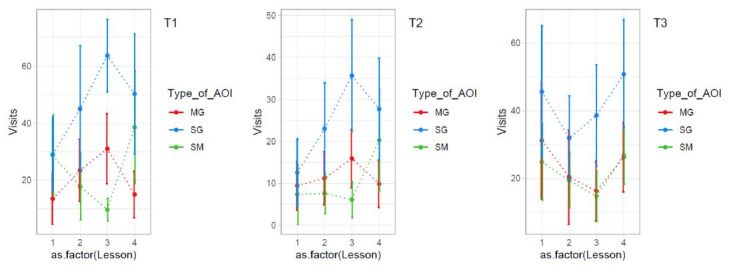
Visits distribution for mutual gaze, single gaze and student material for each teacher and each lesson.

There is a possibility that visits to mutual gaze, single gaze, and student material vary over the four lessons for all three teachers. These data are plotted in Figure 7 which indicates that teacher 1 and teacher 2 have a similar development of behaviour over lessons. Figure 7 also suggests a trade-off in the number of visits between the single gaze and student material gaze. In contrast, teacher 3 appears to behave entirely differently.

The large variation in Figure 7 is puzzling, but probably reflects a variety of teaching behaviour across the lessons. To quantify whether such a variety across lessons exists also for other factors, we calculated the same kind of plots for visits depending on gender, achievement, and T-Zone. We then calculated the variety across lessons using standard errors of variety.

In the left part of Table 5, the standard errors of the nine plots in Figure 7 are shown. Note how the standard errors correspond to the variety across lessons observed in Figure 7. The remaining part of Table 5 shows standard errors for the other factors. We can see that the highest variety in visits across lessons was observed for the student material category and the outside of the T-Zone category.

Again, this does not apply to teacher 3, only to teacher 1 and teacher 2. Teacher 3 is demonstrating less varied behavior.

**Table 5:** Visits: Standard errors of variety across four lessons.

VISITS	T1	T2	T3	MEAN
MG	0.188	0.094	0.304	0.195
SG	0.295	0.203	0.184	0.227
SM	0.527	0.501	0.184	**0.404**
F	0.275	0.279	0.185	0.247
M	0.150	0.353	0.262	0.255
High	0.204	0.324	0.190	0.240
Low	0.133	0.247	0.286	0.222
IN	0.173	0.301	0.237	0.237
OUT	0.310	0.370	0.227	**0.303**
MEAN	0.251	0.297	0.229	

### Data exploration for follow-up research

Since the data indicated great variety in single gaze, mutual gaze and student material gaze over the 4 lessons, we decided to also explore possible variation within the lesson to illustrate the tentative reasons for the differences. A 45-minute lesson can be expected to be structured into small units that may be very different in-between them. As an example, we have plotted data from teacher 1, who had the largest variety across lessons of gaze on student material (Table 5). We have applied a 5 second averaging filter to the raw AOI over time data to produce these plots.

**Figure 8a:**
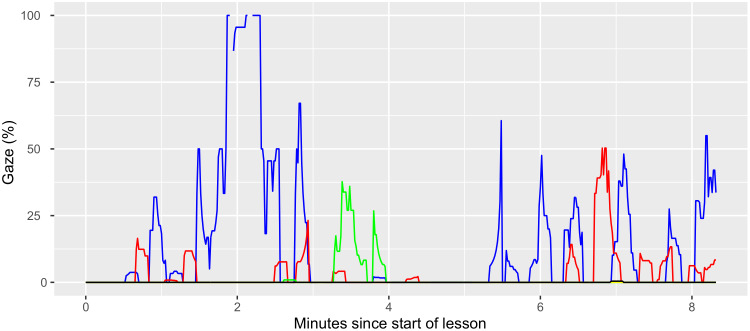
At the start of the lesson.

In Figure 8a, we can see that teacher 1 starts the lesson with many SG gazes on pupils (blue), with very few MG gazes (red). Scanning the class at the start of the lesson is concluded by briefly gazing at student material (green). After a 1-minute break, teacher 1 starts the next activity, which again features SG gazes but now with more frequent and longer mutual (MG) gazes.

**Figure 8b:**
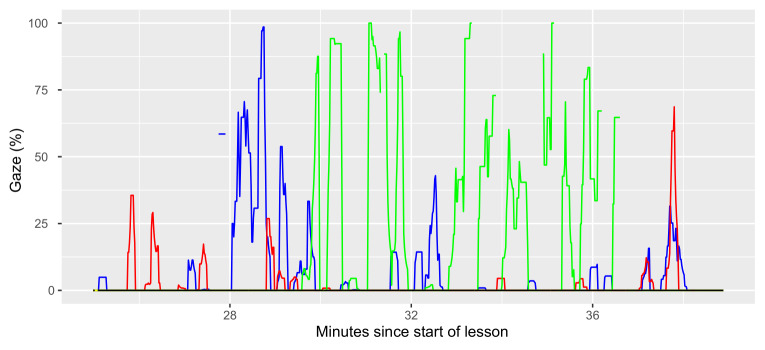
Activity during the period 26-38 minutes into the lesson.

After about half an hour of activity spurts that involve MG and SG, teacher 1 spends six minutes almost exclusively reading student material (Figure 8b). In this recording, time spent reading student material is not equally distributed over the lesson. SM gazes are focused to a few very concentrated periods, during which the long dwell times of Figure 6 are produced. SG and MG are much more equally distributed over time. This also explains the results in Figure 5 that we see fewer but longer SM compared to SG when we average over the whole lessons.

## Discussion

We have explored the distribution of gaze of three experienced teachers in their real-world classrooms using mobile eye-tracking technology during 12 complete lessons of English as a foreign language. Results show substantial variation in gaze distribution between teachers, and between individual lessons of each teacher, as well as inequality of gaze distribution towards individual students. In the following sections, we summarize and discuss the key findings.

### Variety in the teachers' gaze distribution

There is a large gap between the number of times a teacher gazes on the most and least watched students in the classroom, with the most watched students receiving 3-8 times more gaze (around 80 more looks). This is an extreme difference and it raises a serious question as to whether the teacher should be considered as unfair in their attention distribution towards their students, or whether this variation represents an adaptation to a specific means of managing the classroom, or there is a different underlying cause.

The results from calculated GINI coefficients demonstrated that the gaze towards all students in the classroom was distributed unequally, which suggests that some students or groups of students obtain more attention than others. The GINI coefficient of values for visits were similar among all three teachers (0.36; 0.37; 0.31). GINI coefficients were lower for average dwell time, which suggests that teachers are fairer with how long they look at students, and more unfair with how many times they look.

Previous studies used GINI coefficient with amalga-mated measures. Cortina et al. (5) reported GINI coefficients for the number of fixations per students in a 45-min class period, finding for expert teachers (n = 12) the mean value 0.27 and for novice teachers (n = 12) 0.34 (compare to our participants’ values of 0.41; 0.43; 0.33). Dessus et al. (6) calculated their GINI coefficients from the Relative total dwell time (amalgamation of visits with dwell time) per students in a 45-min class period, and found for expert teachers (n = 2) the mean value 0.35 and 0.29 and novice teachers (n = 2) 0.33 and 0.32, compared with our teacher participants at 0.41, 0.44 and 0.34.

Our conclusion is that so far it has not been consistently shown that gaze distribution becomes more equal with more experience.

### Gaze distribution with respect to the three characteristics of the students

Previous classroom interaction studies have repeatedly found that more attention goes towards boys than towards girls (21, 22, 23), to the high achievers more often than to the low achievers and, finally, more attention goes towards the students sitting in the T-Zone than towards those sitting outside the T-Zone. In contrast, we found no effects on the factor of gender. It is unclear why, but we note that it has been shown that the professional vision of teachers of science and mathematics is different from the professional vision of teachers of social sciences and the humanities domain (112). It is possible that teachers in the English as a second language class gaze equally towards boys and girls and if we had measured three science teachers, we would have seen a difference based on gender. Another explanation could be a change in teacher perception of gender: The majority of previous studies showing that teachers pay more attention to boys were made in the 20th century when gender differences may have been more strongly expected by teachers than they are today, which would guide their gaze differently. In any case, the gender question deserves a deeper review of a larger sample of teachers across subjects and possibly also in diverse cultural contexts.

Similarly as in the case of gender, in contrast to previous studies, we found no effect on teacher gaze for the factor of student achievement.

However, for the students sitting in the T-Zone, teachers made about 5 more visits (t=-5.0; p<0.001)) per lesson and student, compared to those sitting outside this zone. This is in line with many previous results showing that teachers tend to look more at students sitting at certain places in the traditional classroom seating arrangement (43, 44, 45, 46, 48, 49, 50, 52, 53).

Since the linear regression of visits signaled signifi-cant effects only for the factor of position in the classroom (besides teacher and type of AOI), among our three selected factors, and the explained variance is relatively low (R^2^ = 0.40), there must be other underlying factors causing this variation in where teachers choose to place their gaze. These could include personality characteristics of the teachers, progression of the teaching activity over a lesson, or between lessons. The findings can also be interpreted in the context of teacher expertise, in that expert teachers act in the classroom concerning the whole personality of the student and the actual practice of the teacher is predominantly oriented on a long-term period that goes beyond a particular lesson (113).

Alternatively, taking into account the relatively high GINI coefficients mentioned previously, and the influence of classroom position on gaze distribution, we can ask whether they are a sign of lack of quality teaching among our participants, despite their experience. Further gaze distribution studies conceptualizing teaching quality and expertise beyond years of experience could help us understand which of the alternative explanations for our findings is more feasible.

### Frequency and duration of mutual gaze, single gaze, and gaze on student material

Our data show that all three teachers made on average 18 more single gazes per student during each lesson than mutual gazes and student material gazes. The number of mutual gazes and gazes at the student material was about the same. These findings are in line with previous studies that show student-centered mentality of teachers (114) with prevalence of single gaze (8). This could be caused by multiple functions of the gaze in naturalistic classrooms. One is checking where the teacher is looking around at the students inspecting who is paying attention and searching for signs of students being bored or lost (63, 64). Mostly during inspections, students will not be looking in the direction of the teacher, and there is only the odd accidental mutual gaze. Other functions of teachers’ gaze are to support students who are currently talking (69), or to use gaze as a sign of approval or disapproval (74). In these two functions, a single gaze and mutual gaze could both be used, with proportions that reflect the needs of the situation. A third reason for the dominance of single gaze over mutual gaze is the intentional gaze aversion that students do when they do not know the answer to the question (96); when answering cognitively challenging questions (1); or gaze aversion by students with low self-esteem (98, 99).

When it comes to gaze duration, we found that single and mutual gaze both have short dwell times (about 500 ms on average). In experimental laboratory research, participants tend to terminate mutual gazes earlier than single gazes (115), but in our classroom data, this was not the case. Moreover, on average, the longest gaze was on student material areas. This contrasts with the results of McIntyre’s et al. (8) study of expert teachers. It is important to note, however, that in our data, longer average gaze durations were caused by some very long dwell times (up to 5500 ms) recorded when teachers provided intervention in connection with student materials. The underlying function of each form of gaze is vital in interpreting the outcomes in the different eye-movement metrics.

### Variety in single gaze, mutual gaze and student material gaze over the 4 lessons

Our analyses of gaze over consecutive lessons show that both teacher 1 and teacher 2 are utilizing a trade-off strategy between the single gaze and student material gaze: More visits by single gaze covaries with fewer visits to student material and vice versa. Teacher 3 behaved differently, however, with no obvious co-variation and a more stable behaviour across lessons.

The highest variation, measured as standard error, in the number of visits to AOIs across lessons was observed for the student material and the outside of the T-Zone categories. Variation for the student material category can be caused by varying activities and tasks when the students need to work with their material during each lesson. Variation for the outside of the T-Zone category might be because mostly low achieving students were sitting inside the T-Zone area, leaving the majority of higher achieving students and few low achievers to have a position outside the T-Zone area. Low achievers may need constant amount of attention throughout all the lessons in contrast with higher achieving students, so the area inside the T-Zone could be more stable with more visits (as it was displayed in Figure 4.(c)) with more teachers’ attention contrary to the area outside of the T-Zone where the attention may vary more. In general, teachers arrange the teaching processes (incl. sitting order) according to expectations on students – teachers create their own “diagnosis” of each student (116) to prevent inappropriate behaviour.

Again, this does not apply to teacher 3, who demonstrated a less varied behaviour. We note that teacher 3 has the shortest teaching experience (6 years) compared to teacher 1 and teacher 2 with 10 and 12 years of teaching experience respectively. According to Píšová et al. (117), one effect of having experience as a teacher is the ability to improvise and adapt to the actual situation in the classroom. Expert teachers can be expected to not act the same way in every lesson, as they respond to each current situation, and changes in the future, and it cannot be expected that teachers with different experiences will have the same patterns in their distribution of gaze.

These data were collected for four consecutive les-sons, which allowed us to study this variety. In many previous studies that collected eye-tracking data about teachers’ visual attention in real-world classrooms, the authors either recorded a few minute-long sequences from a single lesson or one entire lesson, which allowed them to include a larger number of teachers in their samples instead of several lessons (5, 6, 7, 8, 9, 10, 11, 12, 13, 14, 15). Our results show that studies would benefit from recording the same teacher over several lessons.

### Future work

As a further step, it would be interesting to systematically explore how the values – number of visits for boys and girls; low achievers and high achievers; students sitting in and outside of the T-Zone and different types of gaze (single gaze; mutual gaze and student material gaze) change within finer time sections, e.g. during 0.5-minute intervals of the lesson. Changes within the lesson could be based on the different activities and tasks happening during the lesson, teacher-student interaction sequences, as well as the natural flow when different patterns could be expected for the beginning, main part and last minutes of the lesson when the students may be tired and less attentive. Figures 8a and 8b provided us a first glimpse of what this kind of analysis could look like.

As a part of the study, we highlighted the importance of appropriate metric selection (visits, dwell time, number of fixations etc.). Our analyses showed that using different metrics yields different perspectives on gaze distribution and thus on teachers’ visual attention in general. Further discussions on the best fitting metric should ensue.

Lastly, gaze distribution as a proxy of teaching quality should be further explored. Findings of this and previous studies provide different results on gaze distribution of beginning, experienced, and expert teachers. Studies using eye movement metrics in combination with thorough conceptualization of teacher expertise would be beneficial.

### Ethics and Conflict of Interest

The author(s) declare(s) that the contents of the article are in agreement with the ethics described in http://biblio.unibe.ch/portale/elibrary/BOP/jemr/ethics.html and that there is no conflict of interest regarding the publication of this paper.

### Acknowledgements

This work was supported by the project English teachers’ professional vision in/on action in communicative activities from the perspective of eye tracking (GA17-15467S) funded by the Czech Science Foundation and by the research infrastructure HUME Lab Experimental Humanities Laboratory, Faculty of Arts, Masaryk University.

## References

[b43] Adams, R., & Biddle, B. (1970). The classroom scene. Realities of teaching. Holt, Rinehart & Winston.

[b86] Andersen, J. F. (1979). Teacher Immediacy as a Predictor of Teaching Effectiveness. Annals of the International Communication Association, 3(1), 543–559. 10.1080/23808985.1979.11923782 2380-8985

[b23] Aukrust, V. G. V. (2008). Boys’ and girls’ conversational participation across four grade levels in Norwe-gian classrooms: Taking the floor or being given the floor? Gender and Education, 20(3), 237–252. 10.1080/09540250802000413 0954-0253

[b110] Bellù, J. G., & Liberati, P. Describing income in-equality: Theil index and entropy class indexes. FAO EASYPol. 2006;051. Available from: http://www.fao.org/docs/up/easypol/445/theil_index_051en.pdf

[b103] Birmingham, E., Bischof, W. F., & Kingstone, A. (2009). Get real! Resolving the debate about equivalent so-cial stimuli. Visual Cognition, 17(6-7), 904–924. 10.1080/13506280902758044 1350-6285

[b112] Blomberg, G., Stürmer, K., & Seidel, T. (2011). How pre-service teachers observe teaching on video: Ef-fects of viewers’ teaching subjects and the sub-ject of the video. Teaching and Teacher Education, 27(7), 1131–1140. 10.1016/j.tate.2011.04.008 0742-051X

[b44] Breed, G., & Colaiuta, V. (1974). Looking, blinking, and sitting: Nonverbal dynamics in the classroom. Journal of Communication, 24(2), 75–81. 10.1111/j.1460-2466.1974.tb00371.x 0021-9916 4823233

[b38] Brophy, J. E., & Good, T. L. (1970). Teachers communication of differential expectations for childrens class-room performance: Some behavioral data. Journal of Educational Psychology, 61(5), 365–374. 10.1037/h0029908 0022-0663

[b76] Caproni, V., Levine, D., Oneal, E., Mcdonald, P., & Garwood, G. (1977). Seating Position, Instructors Eye Contact Availability, and Student Participation in a Small Seminar. The Journal of Social Psychology, 103(2), 315–316. 10.1080/00224545.1977.9713335 0022-4545

[b78] Cary, M. S. (1978). The Role of Gaze in the Initiation of Conversation. Social Psychology, 41(3), 269-271. 10.2307/3033565 0147-829X

[b41] Chaikin, A. L., Sigler, E., & Derlega, V. J. (1974). Nonverbal mediators of teacher expectancy effects. Journal of Personality and Social Psychology, 30(1), 144–149. 10.1037/h0036738 0022-3514

[b94] Chen, F. S., Minson, J. A., Schöne, M., & Heinrichs, M. (2013). In the eye of the beholder: Eye contact increases resistance to persuasion. Psychological Science, 24(11), 2254–2261. 10.1177/0956797613491968 0956-7976 24068114

[b5] Cortina, K. S., Miller, K. F., Mckenzie, R., & Epstein, A. (2015). Where Low and High Inference Data Converge: Validation of CLASS Assessment of Mathemat-ics Instruction Using Mobile Eye Tracking with Expert and Novice Teachers. International Journal of Science and Mathematics Education, 13(2), 389–403. 10.1007/s10763-014-9610-5 1571-0068

[b87] Csibra, G., & Gergely, G. (2009). Natural pedagogy. Trends in Cognitive Sciences, 13(4), 148–153. 10.1016/j.tics.2009.01.005 1364-6613 19285912

[b58] DeWall, C. N., & Maner, J. K. (2008). High Status Men (but Not Women) Capture the Eye of the Beholder. Evolutionary Psychology, 6(2), 147470490800600. 10.1177/147470490800600209 1474-7049

[b6] Dessus, P., Cosnefroy, O., & Luengo, V. (2016). “Keep Your Eyes on ‘em all!”: A Mobile Eye-Tracking Analysis of Teachers’ Sensitivity to Students. Adaptive and Adaptable Learning Lecture Notes in Computer Science.

[b102] Doherty-Sneddon, G., Bruce, V., Bonner, L., Longbotham, S., & Doyle, C. (2002). Development of gaze aversion as disengagement from visual information. Developmental Psychology, 38(3), 438–445. 10.1037/0012-1649.38.3.438 0012-1649 12005386

[b1] Doyle, W. (1977). Learning the Classroom Environment: An Ecological Analysis. Journal of Teacher Education, 28(6), 51–55. 10.1177/002248717702800616 0022-4871

[b31] Erden, F., & Wolfgang, C. (2004). An exploration of the differences in prekindergarten, kindergarten, and first grade teachers’ beliefs related to discipline when dealing with male and female students. Early Child Development and Care, 174(1), 3–11. 10.1080/0300443032000103098 0300-4430

[b65] Ergin, A., & Birol, C. (2005). Eğitimde iletişim. Anı Yayıncılık.

[b91] Exline, R. V., & Winters, L. C. (1965). Affective relations and mutual glances in dyads. In S. Tomkins & C. Izzard (Eds.), Affect, Cognition and Personality (pp. 319–330). Springer International Publishing.

[b79] Falck-Ytter, T., Thorup, E., & Bölte, S. (2015). Brief report: Lack of processing bias for the objects other people attend to in 3-year-olds with autism. Journal of Autism and Developmental Disorders, 45(6), 1897–1904. 10.1007/s10803-014-2278-4 0162-3257 25331324 PMC4441907

[b55] Feldman, R. S. (1976). Nonverbal disclosure of teacher deception and interpersonal affect. Journal of Educational Psychology, 68(6), 807–816. 10.1037/0022-0663.68.6.807 0022-0663

[b59] Foulsham, T., Cheng, J. T., Tracy, J. L., Henrich, J., & Kingstone, A. (2010). Gaze allocation in a dynamic situation: Effects of social status and speaking. Cognition, 117(3), 319–331. 10.1016/j.cognition.2010.09.003 0010-0277 20965502

[b29] Francis B. Boys, girls, and achievement ad-dressing the classroom issues. London: Routled-geFalmer; 2000.

[b75] Fry, R., & Smith, G. F. (1975). The Effects of Feedback and Eye Contact on Performance of a Digit-Coding Task. The Journal of Social Psychology, 96(1), 145–146. 10.1080/00224545.1975.9923275 0022-4545

[b70] Frymier, A. B. (1994). A model of immediacy in the classroom. Communication Quarterly, 42(2), 133–144. 10.1080/01463379409369922 0146-3373

[b105] Gallup, A. C., Hale, J. J., Sumpter, D. J. T., Garnier, S., Kacelnik, A., Krebs, J. R., & Couzin, I. D. (2012). Visual attention and the acquisition of information in human crowds. Proceedings of the National Academy of Sciences of the United States of America, 109(19), 7245–7250. 10.1073/pnas.1116141109 0027-8424 22529369 PMC3358867

[b35] Garrahy, D. A. (2001). Three Third-Grade Teachers Gen-der-Related Beliefs and Behavior. The Elementary School Journal, 102(1), 81–94. 10.1086/499694 0013-5984

[b60] Gerpott, F. H., Lehmann-Willenbrock, N., Silvis, J. D., & Vugt, M. V. (2018). In the eye of the beholder? An eye-tracking experiment on emergent leadership in team interactions. The Leadership Quarterly, 29(4), 523–532. 10.1016/j.leaqua.2017.11.003 1048-9843

[b66] Gibson, J. J., & Pick, A. D. (1963). Perception of another person’s looking behavior. The American Journal of Psychology, 76(3), 386–394. 10.2307/1419779 0002-9556 13947729

[b100] Glenberg, A. M., Schroeder, J. L., & Robertson, D. A. (1998). Averting the gaze disengages the environment and facilitates remembering. Memory & Cognition, 26(4), 651–658. 10.3758/BF03211385 0090-502X 9701957

[b39] Good, T. L., Sikes, J. N., & Brophy, J. E. (1973). Effects of teacher sex and student sex on classroom inter-action. Journal of Educational Psychology, 65(1), 74–87. 10.1037/h0034816 0022-0663

[b27] Good, T. L., Cooper, H. M., & Blakey, S. L. (1980). Classroom interaction as a function of teacher expectations, student sex, and time of year. Journal of Educational Psychology, 72(3), 378–385. 10.1037/0022-0663.72.3.378 0022-0663

[b26] Good, T. L., & Findley, M. J. (1985). Sex role expectations and achievement. In J. B. Dusek (Ed.), Teacher ex-pectations (pp. 32–56). Erlbaum.

[b40] Good, T. L. (1997). Looking in classrooms. Longman.

[b69] Gower, R., & Walters, S. (1983). Teaching practice hand-book: a reference book for EFL teachers in training. Heinemann.

[b88] Hamlet, C. C., Axelrod, S., & Kuerschner, S. (1984). Eye contact as an antecedent to compliant behavior. Journal of Applied Behavior Analysis, 17(4), 553–557. 10.1901/jaba.1984.17-553 0021-8855 16795682 PMC1307977

[b98] Hartley, G., & Karinch, M. (2007). I Can Read You Like a Book: How to Spot the Messages and Emotions People Are Really Sending With Their Body Language. Career Press.

[b116] Hawkins, D. (2002). The informed vision: essays on learning and human nature. Algora Pub.

[b115] Helminen, T. M., Kaasinen, S. M., & Hietanen, J. K. (2011). Eye contact and arousal: The effects of stimulus duration. Biological Psychology, 88(1), 124–130. 10.1016/j.biopsycho.2011.07.002 0301-0511 21782881

[b89] Hietanen, J. K., Leppänen, J. M., Peltola, M. J., Linna-Aho, K., & Ruuhiala, H. J. (2008). Seeing direct and averted gaze activates the approach-avoidance motivational brain systems. Neuropsychologia, 46(9), 2423–2430. 10.1016/j.neuropsychologia.2008.02.029 0028-3932 18402988

[b61] Holland, E., Wolf, E. B., Looser, C., & Cuddy, A. (2017). Visual attention to powerful postures: People avert their gaze from nonverbal dominance displays. Journal of Experimental Social Psychology, 68, 60–67. 10.1016/j.jesp.2016.05.001 0022-1031

[b109] Holmqvist, K., & Andersson, R. (2017). Eye tracking: a comprehensive guide to methods, paradigms, and measures. Oxford University Press.

[b16] Howe, C., & Abedin, M. (2013). Classroom dialogue: A sys-tematic review across four decades of research. Cambridge Journal of Education, 43(3), 325–356. 10.1080/0305764X.2013.786024 0305-764X

[b63] Huang, L. (2011). Nonverbal Communication in College English Classroom Teaching. Journal of Language Teaching and Research., 2(4), 903 908. 10.4304/jltr.2.4.903-908

[b28] Irvine, J. J. (1986). Teacher–student interactions: Effects of student race, sex, and grade level. Journal of Educational Psychology, 78(1), 14–21. 10.1037/0022-0663.78.1.14 0022-0663

[b22] Jones, S. M., & Dindia, K. (2004). A Meta-Analytic Perspec-tive on Sex Equity in the Classroom. Review of Educational Research, 74(4), 443–471. 10.3102/00346543074004443 0034-6543

[b42] Jong, C. (2017). Extending Equitable Practices in Teach-er Noticing: Commentary. Teacher Noticing: Bridging and Broadening Perspectives. Contexts, and Frameworks.

[b57] Just, M. A., & Carpenter, P. A. (1980). A theory of reading: From eye fixations to comprehension. Psychological Review, 87(4), 329–354. 10.1037/0033-295X.87.4.329 0033-295X 7413885

[b80] Kelley, D. H., & Gorham, J. (1988). Effects of immediacy on recall of information. Communication Education, 37(3), 198–207. 10.1080/03634528809378719 0363-4523

[b21] Kelly, A. (1988). Gender differences in teacher–pupil in-teractions: A meta-analytic review. Research in Education, 39(1), 1–23. 10.1177/003452378803900101 0034-5237

[b71] Kerssen-Griep, J., & Witt, P. L. (2012). Instructional Feed-back II: How Do Instructor Immediacy Cues and Facework Tactics Interact to Predict Student Motivation and Fairness Perceptions? Communication Studies, 63(4), 498–517. 10.1080/10510974.2011.632660 1051-0974

[b7] Kim WJ, Beyon JH, Lee IS, Kwon YJ. Gaze dif-ferences between expert and novice teachers in science classes. 한국과학교육학회지. 2012;32(9):1443-51.

[b108] Kingstone, A., Smilek, D., & Eastwood, J. D. (2008). Cognitive Ethology: A new approach for studying human cognition. British Journal of Psychology, 99(Pt 3), 317–340. 10.1348/000712607X251243 0007-1269 17977481

[b113] Kirshner, D., & Whitson, J. A. (1998). Obstacles to Under-standing Cognition As Situated. Educational Researcher, 27(8), 22–28. 10.3102/0013189X027008022 0013-189X

[b92] Kleinke, C. L. (1986). Gaze and eye contact: A research review. Psychological Bulletin, 100(1), 78–100. 10.1037/0033-2909.100.1.78 0033-2909 3526377

[b96] Knapp, M. L., & Hall, J. A. Nonverbal communication in human interaction. Harcourt, Brace, Jo-vanovich: Fort Worth; 1992.

[b106] Laidlaw, K. E. W., Foulsham, T., Kuhn, G., & Kingstone, A. (2011). Potential social interactions are important to social attention. Proceedings of the National Academy of Sciences of the United States of America, 108(14), 5548–5553. 10.1073/pnas.1017022108 0027-8424 21436052 PMC3078350

[b64] Ledbury, R., White, I., & Darn, S. (2004, 8). The importance of eye contact in the classroom. The Internet TESL Journal., 10(8), 11–21.

[b68] Manen, M. V. (2012). The Call of Pedagogy as the Call of Contact. Phenomenology & Practice, 6(2), 8–34. 10.29173/pandpr19859 1913-4711

[b62] Maner, J. K., DeWall, C. N., & Gailliot, M. T. (2008). Selective attention to signs of success: Social dominance and early stage interpersonal perception. Personality and Social Psychology Bulletin, 34(4), 488–501. 10.1177/0146167207311910 0146-1672 18192434

[b45] Marx, A., Fuhrer, U., & Hartig, T. (1999, 10). Effects of class-room seating arrangements on children’s ques-tionasking. Learning Environments Research, 2(3), 249–263. 10.1023/A:1009901922191 1387-1579

[b46] McCorskey, J. C., & McVetta, R. W. (1978). Classroom seat-ing arrangements: Instructional communication theory versus student preferences. Communication Education, 27(2), 99–111. 10.1080/03634527809378281 0363-4523

[b84] McCroskey, J. C., Richmond, V. P., Sallinen, A., Fayer, J. M., & Barraclough, R. A. (1995). A cross-cultural and multi-behavioral analysis of the relationship between nonverbal immediacy and teacher evaluation. Communication Education, 44(4), 281–291. 10.1080/03634529509379019 0363-4523

[b9] McIntyre, N. A., Mainhard, M. T., & Klassen, R. M. (2017). Are you looking to teach? Cultural, temporal and dynamic insights into expert teacher gaze. Learning and Instruction, 49, 41–53. 10.1016/j.learninstruc.2016.12.005 0959-4752

[b10] McIntyre, N. A., & Foulsham, T. (2018, 1 5). Scanpath analysis of expertise and culture in teacher gaze in real-world classrooms. Instructional Science, 46(3), 435–455. 10.1007/s11251-017-9445-x 0020-4277

[b8] McIntyre, N. A., Jarodzka, H., & Klassen, R. M. (2019). Captur-ing teacher priorities: Using real-world eye-tracking to investigate expert teacher priorities across two cultures. Learning and Instruction, 60, 215–224. 10.1016/j.learninstruc.2017.12.003 0959-4752

[b14] McIntyre, N. A., Mulder, K. T., & Mainhard, M. T. (2020). Looking to relate: Teacher gaze and culture in student-rated teacher interpersonal behaviour. Social Psychology of Education, 23, 411–431. 10.1007/s11218-019-09541-2 1381-2890

[b24] Measor, L., & Sikes, P. J. (1992). Gender and education. Cassell & Mansell.

[b2] Mitchell, R. N., & Marin, K. A. (2015, 12 10). Examining the use of a structured analysis framework to support pros-pective teacher noticing. Journal of Mathematics Teacher Education, 18(6), 551–575. 10.1007/s10857-014-9294-3 1386-4416

[b47] Montello, D. R. (1988, 6). Classroom seating location and its effect on course achievement, participation, and attitudes. Journal of Environmental Psychology, 8(2), 149–157. 10.1016/S0272-4944(88)80005-7 0272-4944

[b111] Mussard, S., Seyte, F., & Terraza, M. (2003). Decomposition of Gini and the Generalized Entropy Inequality Measures. Economic Bulletin, 4(7), 1–6.0343-754X

[b32] Myhill, D. (2002). Bad Boys and Good Girls? Patterns of Interaction and Response in Whole Class Teach-ing. British Educational Research Journal, 28(3), 339–352. 10.1080/01411920220137430 0141-1926

[b95] Neill, S. R., & Caswell, C. (1993). Body language for compe-tent teachers. Routledge.

[b67] Noddings, N. (2003). Happiness and education. Cam-bridge. Cambridge University Press. 10.1017/CBO9780511499920

[b33] Ohrn, E. (1993). Gender, Influence and Resistance in School British Journal of Sociology of Education, 14(2), 147–158. 10.1080/0142569930140202 0142-5692

[b74] Otteson, J. P., & Otteson, C. R. (1980). Effect of Teachers Gaze on Children’s Story Recall. Perceptual and Motor Skills, 50(1), 35–42. 10.2466/pms.1980.50.1.35 0031-5125

[b99] Pease, A., & Pease, B. The definitive book of body language (Bantam hardcover ed.): The hidden meaning behind people's gestures and expres-sions. New York: Bantam; 2006.

[b77] Pedersen, D. M. (1977). Relationship of Ratings of Class-room Performance and Enjoyment with Seat Se-lection. Perceptual and Motor Skills, 45(2), 601–602. 10.2466/pms.1977.45.2.601 0031-5125

[b48] Pedersen, D. M. (1994). Personality and Classroom Seat-ing. Perceptual and Motor Skills, 78(3, suppl), 1355–1360. 10.2466/pms.1994.78.3c.1355 0031-5125

[b101] Phelps, F. G., Doherty-Sneddon, G., & Warnock, H. (2006). Helping children think: Gaze aversion and teaching. British Journal of Developmental Psychology, 24(3), 577–588. 10.1348/026151005X49872 0261-510X

[b83] Pollitt, L. Classroom Management. Tesol Course Articles; 2006 [cited 2020 Jan20]. Available from: http://www.tesolcourse.com

[b11] Prieto, L. P., Sharma, K., & Dillenbourg, P. (2015). Studying teacher orchestration load in technology-enhanced classrooms. In G. Conole, T. Klobučar, Ch. Rensing, J. Konert, & E. Lavoué (Eds.), Design for teaching and learning in a networked world (pp. 268–281). Springer International Publishing. 10.1007/978-3-319-24258-3_20

[b117] Píšová M, Hanušová S, Kostková K, Janíková V, Najvar P, Tůma F. Učitel expert: jeho charak-teristiky a determinanty profesního rozvoje (na pozadí výuky cizích jazyků). Brno: Masarykova univerzita; 2012.

[b36] Raider-Roth, M. B., Albert, M. K., Bircann-Barkey, I., Gidseg, E., & Murray, T. (2008). Teaching boys: A relation-al puzzle. Teachers College Record, 110(2), 443–481.0040-0475

[b72] Richmond, V. P., Gorham, J. S., & McCroskey, J. C. (1987). The relationship between selected immediacy beha-viors and cognitive learning. In M. L. Mclaughlin (Ed.), Communication yearbook 10 (pp. 574–590). Sage.

[b107] Risko, E. F., Richardson, D. C., & Kingstone, A. (2016). Breaking the Fourth Wall of Cognitive Science. Current Directions in Psychological Science, 25(1), 70–74. 10.1177/0963721415617806 0963-7214

[b90] Rosenthal, R., & Jacobson, L. (1966). Teachers’ expectancies: Determinants of pupils’ IQ gains. Psychological Reports, 19(1), 115–118. 10.2466/pr0.1966.19.1.115 0033-2941 5942071

[b25] Sadker, M., Sadker, D., & Zittleman, K. R. (2009). Still failing at fairness: How gender bias cheats girls and boys in school and what we can do about it. Simon and Schuster.

[b3] Santagata, R. (2011). From teacher noticing to a frame-work for analyzing and improving classroom lessons. In M. S. Sherin, V. R. Jacobs, & R. A. Philipp (Eds.), Mathematics teacher noticing (pp. 152–168). Routledge.

[b49] Schnitzerová, E., & Račková, E. (1995). Niektoré kvanti-tatívne charakteristiky pedagogickej interakcie. Psychológia a Patopsychológia Dieťaťa, 3, 293–301.0555-5574

[b50] Schwebel, A. I., & Cherlin, D. L. (1972). Physical and social distancing in teacher-pupil relationships. Journal of Educational Psychology, 63(6), 543–550. 10.1037/h0034081 0022-0663

[b93] Segrin, C. (1993). The effects of nonverbal behavior on outcomes of compliance gaining attempts. Communication Studies, 44(3–4), 169–187. 10.1080/10510979309368393 1051-0974

[b85] Senju, A., & Johnson, M. H. (2009). The eye contact effect: Mechanisms and development. Trends in Cognitive Sciences, 13(3), 127–134. 10.1016/j.tics.2008.11.009 1364-6613 19217822

[b12] Sherin, M. G., Russ, R. S., Sherin, B. L., & Colestock, A. (2008). Professional vision in action: An exploratory study. Issues in Teacher Education, 17(2), 27–46.1536-3031

[b13] Sherin, M. G., Jacobs, V. R., & Philipp, R. A. (Eds.). (2011). Mathematics teacher noticing. Seeing through teachers’ eyes. Routledge. 10.4324/9780203832714

[b81] Sherwood JV. Facilitative Effects of Gaze upon Learning. Perceptual and Motor Skills. 1987;6 4(3_suppl): 1275–8. 10.2466/pms.1987.64.3c.1275

[b104] Smith, T. J., & Mital, P. K. (2013). Attentional synchrony and the influence of viewing task on gaze behavior in static and dynamic scenes. Journal of Vision (Charlottesville, Va.), 13(8), 16. 10.1167/13.8.16 1534-7362 23863509

[b73] Snyder, D. W. (1998). Classroom Management for Stu-dent Teachers. Music Educators Journal, 84(4), 37–40.0027-4321

[b51] Sommer, R. (1967). Classroom Ecology. The Journal of Applied Behavioral Science, 3(4), 489–503. 10.1177/002188636700300404 0021-8863

[b52] Sommer, R. (1969). Personal Space. Prentice-Hall.

[b17] Spender D. Invisible women. The schooling scandal. Trowbridge: Writers and Readers Pub-lishing; 1982.

[b18] Stake, J. E., & Katz, J. F. (1982). Teacher-Pupil Relationships in the Elementary School Classroom: Teacher-Gender and Pupil-Gender Differences. American Educational Research Journal, 19(3), 465–471. 10.3102/00028312019003465 0002-8312

[b54] Stires, L. (1980, 6 1). Classroom Seating Location, Student Grades, and Attitudes. Environment and Behavior, 12(2), 241–254. 10.1177/0013916580122008 0013-9165

[b15] Stürmer, K., Seidel, T., Müller, K., Häusler, J., & Cortina, K. S. (2017). What is in the eye of preservice teachers while instructing? An eye-tracking study about attention processes in different teaching situa-tions. Zeitschrift für Erziehungswissenschaft, 20(S1), 75–92. 10.1007/s11618-017-0731-9 1434-663X

[b53] Totusek, P. F., & Staton-Spicer, A. Q. (1982). Classroom Seat-ing Preference as a Function of Student Perso-nality. Journal of Experimental Education, 50(3), 159–163. 10.1080/00220973.1982.11011818 0022-0973

[b34] Tsouroufli, M. (2002). Gender and Teachers Classroom Practice in a Secondary School in Greece. Gender and Education, 14(2), 135–147. 10.1080/09540250220133996 0954-0253

[b4] Walkoe, J. (2015). Exploring teacher noticing of student algebraic thinking in a video club. Journal of Mathematics Teacher Education, 18(6), 523–550. 10.1007/s10857-014-9289-0 1386-4416

[b30] Warrington, M., & Younger, M. (2000). The Other Side of the Gender Gap. Gender and Education, 12(4), 493–508. 10.1080/09540250020004126 0954-0253

[b97] Waxer, P. H. (1974). Therapist training in nonverbal communication. I: Nonverbal cues for depression. Journal of Clinical Psychology, 30(2), 215–218. 10.1002/1097-4679(197404)30:2<215::AID-JCLP2270300229>3.0.CO;2-Q 0021-9762 4448849

[b82] Witt, P. L., Wheeless, L. R., & Allen, M. (2004). A me-ta‐analytical review of the relationship between teacher immediacy and student learning. Communication Monographs, 71(2), 184–207. 10.1080/036452042000228054 0363-7751

[b114] Wolff, C. E., Jarodzka, H., Bogert, N. V. D., & Boshui-zen, H. P. A. (2016). Teacher vision: Expert and novice teachers’ perception of problematic classroom management scenes. Instructional Science, 44(3), 243–265. 10.1007/s11251-016-9367-z 0020-4277

[b56] WoolFolk, R. L., & Woolfolk, A. E. (1974). Nonverbal Teacher Behaviors: A Rejoinder. American Educational Research Journal, 11(3), 307–309. 10.3102/00028312011003307 0002-8312

[b19] Younger, M., & Warrington, M. (1996). Differential Achievement of Girls and Boys at GCSE: Some observations from the perspective of one school. British Journal of Sociology of Education, 17(3), 299–313. 10.1080/0142569960170304 0142-5692

[b20] Younger, M., Warrington, M., & Williams, J. (1999). The Gender Gap and Classroom Interactions: Reality and rhetoric? British Journal of Sociology of Education, 20(3), 325–341. 10.1080/01425699995290 0142-5692

[b37] Younger, M., Warrington, M., & McLellan, R. (2005). Raising boys’ achievements in secondary school: Issues, dilemmas and opportunities. Open University Press.

